# Pharmacogenes Associated With Suicidal Behavior: Addressing a Potential Therapeutic Window

**DOI:** 10.62641/aep.v54i2.2055

**Published:** 2026-04-15

**Authors:** Janette Andrea Becerra López, Alma Delia Genis Mendoza, Humberto Nicolini

**Affiliations:** ^1^Laboratory of Genomics of Psychiatric and Neurodegenerative Diseases, National Institute of Genomic Medicine, 14610 Mexico City, Mexico; ^2^Postgraduate Specialty Program in Genomic Medicine of Psychiatric Disorders, Faculty of Medicine, National Autonomous University of Mexico, UNAM, 04510 Mexico City, Mexico; ^3^Dr. Juan N. Navarro Children’s Psychiatric Hospital, 14080 Mexico City, Mexico; ^4^National Commission on Mental Health and Addictions, CONASAMA, 10200 Mexico City, Mexico

**Keywords:** pharmacogenomics, suicide, risk

## Abstract

Suicide rates in Mexico have been rising, and because suicidal behavior has a genetic component, several pharmacogenes potentially linked with suicide risk have been investigated. This review aims to summarize articles addressing pharmacogenes, their relationship with suicidal behavior phenotypes, and their role in pharmacological treatment response. Among the identified pharmacogenes, variants in genes such as ATP-binding cassette subfamily B member 1 Gene (*ABCB1*) and FKBP Prolyl Isomerase 5 Gene (*FKBP5*) have been repeatedly observed across suicide attempt and completed suicide phenotypes. With these we could hypothesize that there is a possibility of finding shared genetic mechanisms among suicide phenotypes. When studying the response to treatment, the presence of certain variants may result in reduced drug response, yielding no benefit and possibly worsening symptoms, potentially culminating in suicidal behavior. Moreover, overlapping variants have been identified between suicidal behavior and altered response to psychotropic drugs in pharmacogenes involved in different functional pathways such as neurotransmission, hypothalamic-pituitary-adrenal (HPA) regulation and neuroinflammation, so this combination could lead to an increased genetic vulnerability to suicidal behavior. In summary, although data on pharmacogenes related to suicide exist, further research is required to replicate findings in the Mexican population. The insights presented in this review may support the inclusion of other pharmacogenes or variants in existing pharmacogenomic panels to advance precision medicine approaching suicide prevention.

## Introduction

Suicide is a transdiagnostic condition and a major global public health concern, exceeding mortality rates from malaria, human immunodeficiency virus/acquired immunodeficiency syndrome, breast cancer, warfare, and homicide [1]. In Mexico, suicide rate has risen from 4.9 per 100,000 individuals in 2013 to 6.8 last year [2], this highlights the growing need to improve prevention and treatment strategies.

Given that suicidal behavior has a recognized genetic component, current research continues to explore the relationship between vulnerability of suicidal behavior and certain genetic variants in pharmacogenes. In clinical practice, treatment for patients expressing suicidal behavior usually follows a trial-and-error approach. Where individuals may undergo weeks or months of treatment in hopes of improvement or may discontinue treatment due to ineffectiveness or presence of adverse effects. Pharmacogenomics offers a potential solution for instances of non-response to standard dosages of psychotropic medications, helping identify these cases and be able to address the symptoms in an early way [3].

Therefore, the aim of this study is to investigate pharmacogenes and their variants implicated in each phenotype of suicidal behavior and how these variants influence the response to psychotropic drugs. By identifying overlapping or recurrent variants, this review seeks to highlight potential variants that can be considered in the creation of new pharmacogenomic test panels and, consequently, be able to prescribe precision therapies for patients with suicidal behavior.

This review first contextualizes suicidal behavior within the omics framework, then summarizes the pharmacogenes implicated in suicidal ideation, suicide attempts, and suicide completion, classifying it according to their pharmacokinetic or pharmacodynamic function, alongside their influence on pharmacological treatment response. Finally, it integrates findings across suicidal phenotypes and response to treatment to identify convergent pathways and emphasizes the need to continue with further research to advance precision medicine in mental health.

## Suicidal Behavior

Since 1996, different classifications have been proposed to categorize and understand this phenomenon. Suicidal ideation is understood as an exclusively cognitive event involving thoughts of engaging in suicidal behavior [4]; a suicide attempt is described as a potentially self-injurious behavior with a non-fatal outcome for which there is explicit or implicit evidence that the person intended to die [5]; and suicide refers as death caused by self-directed injurious behavior with intent to die [4].

When studying the prevalence and risk factors of suicidal behavior in 17 countries, including Mexico, the estimated lifetime prevalence was found to be 9.2% for suicidal ideation, 3.1% for suicide planning, and 2.7% for suicide attempts. Among those who experience suicidal ideation, the probability of having ever made a suicide plan is 33.6%, and the probability of having ever attempted suicide is 29% [6].

The risk of suicide is influenced by the interaction of various factors: biological, clinical, physical illnesses, cognitive impairments, and psychiatric disorders, the most common in people who die by suicide being major depressive disorder (MDD), bipolar disorder (BD), substance use disorders, and schizophrenia; genetic factors, psychological traits such as Cluster B personality disorders, social factors like relationship breakdowns, job loss, economic instability, being single, or grief; as well as cultural and environmental influences. Predisposing factors include childhood adversity and precipitating factors such as previous suicidal ideation [7].

Suicidal behavior is associated with neurobiological changes that affect various functional pathways, such as the serotonergic system, the HPA axis, neurotrophic pathways, components of the inflammatory process, and dysregulation in gamma-aminobutyric acid (GABA) and glutamate systems [7].

As for treatment options, clinical practice often relies on trial-and-error methods, where a drug is prescribed based on the psychiatric diagnosis or evidence of anti-suicidal effect. Ideally, patients may improve over weeks or months; however, many discontinue treatment due to a lack of improvement or adverse effects. For antidepressants, between 50% and 60% of patients experience poor response and low remission rates [8].

## Omic Techniques in Suicidal Behavior

Suicidal behavior has a genetic component, and in 2021, the first genome-wide association study (GWAS) of 29,782 cases of suicide attempts was conducted by the International Suicide Genetics Consortium (ISGC). The study identified two genome-wide significant loci: the major histocompatibility complex and an intergenic locus on chromosome 7 [9]. A year later, another GWAS was carried out in a large multi-ancestral cohort of U.S. veterans enrolled in the Million Veteran Program, identifying two pan-ancestral genome-wide significant loci on chromosomes 1 and 20 [10].

In Mexico, a genomic association analysis was conducted in individuals with psychiatric diagnoses and suicide attempts. The study found a significant association with the scavenger receptor class A member 5 (*SCARA5*) gene and a nominal association with the growth hormone secretagogue receptor gene (*GHSR*), the regulator of G protein signaling 10, and the serine/threonine kinase 33 [11].

In terms of suicidal ideation, it wasn’t until 2023 that the first GWAS on suicidal ideation without attempt was conducted within the Million Veteran Program cohort. This study identified four genome-wide significant (GWS) loci in the pan-ancestral meta-analysis: on chromosome 2, chromosome 6 with the estrogen receptor 1 (*ESR1*) gene, chromosome 9 with the exonuclease 3^′^-5^′^ domain-containing 3 (*EXD3*) gene, and chromosome 16 with the FBXL19 Antisense RNA 1. The pan-ancestral genetic analysis also revealed GWS associations with the following genes: dopamine receptor D2, deleted in colorectal carcinoma netrin 1 receptor, F-box and leucine-rich repeat protein 19, BAF Chromatin Remodeling Complex Subunit BCL7C, cardiotrophin 1, ankyrin repeat and kinase domain-containing 1, and *EXD3* [12].

Within pharmacogenomics (PGx), it is estimated that additive effects of common genetic polymorphisms in the human genome explain approximately 42% of the individual variation in antidepressant response [13]. Therefore, pharmacogenes could partially explain suicidal events linked to the ineffectiveness of standardized pharmacological treatments in individuals vulnerable to suicide [3].

In 2022, was published the Precision Medicine in Mental Health Care Trial, a randomized controlled trial including 1944 patients with MDD, where with the pharmacogenomic test results select a treatment with fewer potential drug-gene interactions and have greater rates of remission in the group being tested by PGx. The study concluded that the prescription of drugs with interactions was reduced but the effects on remission are small and non-sustained [14].

## Methodology

### Gene Selection Criteria

Initially, an exploratory literature search was conducted to identify pharmacogenes potentially associated with suicidal behavior. It was performed in PubMed and Google Scholar databases between March 5, 2025, and March 25, 2025. The search included general terms such as “pharmacogenomics AND suicide” or “pharmacogenes AND suicide”. We found a wide variety of pharmacogenes, so based on previous psychiatric literature and focusing on genes that are not currently included in pharmacogenomic testing, a targeted list of genes was selected. Three genes that are currently part of the tests, *ABCB1* and two cytochromes, were contemplated.

### Bibliographic Search Strategy

**Fig. 1.**
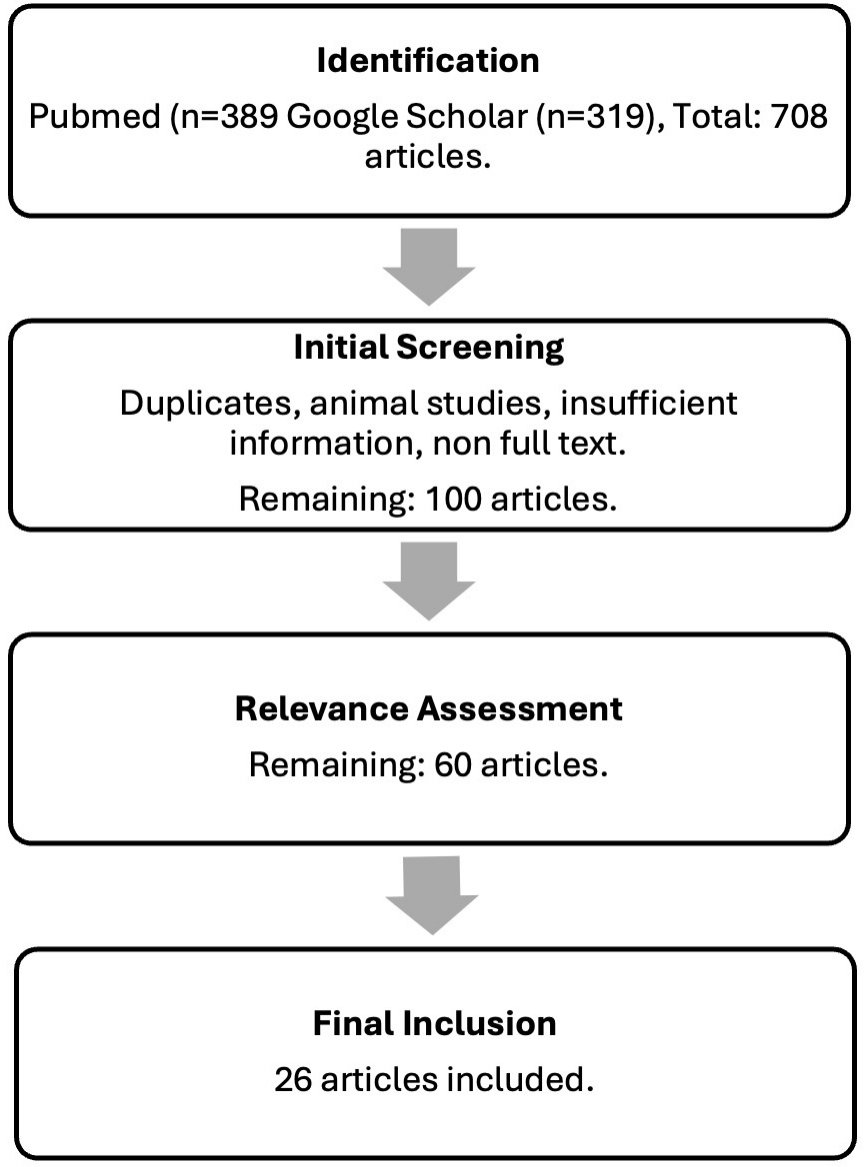
**Flowchart of the literature search and selection process**.

The literature search and selection process are represented using a flowchart adapted from the PRISMA 2020 model, suitable for narrative reviews (Fig. 1). A second, more focused search was then conducted in the databases mentioned above and it was performed between March 30, 2025, and June 25, 2025, and the final update was completed on June 30, 2025. Searches were conducted for each selected pharmacogene individually and combined with suicide-related terms. The overall search strategy was defined using the following Boolean structure (“*ABCB1*” OR “*CYP2D6*” OR “*CYP2C19*” OR “*SCARA5*” OR “*GHSR*” OR “*FKBP5*” OR “*SAT1*” OR “*CRHR1*” OR “*NR3C1*”) AND (“suicide” OR “suicide ideation” OR “suicidal behavior” OR “suicide attempt”).

To explore associations with psychotropic drugs, an additional drug-centered search was conducted in the same databases during the same search period March 30, 2025, and June 25, 2025. Since suicide is a transdiagnostic condition and cannot be confined to a single type of treatment, psychotropic medications from different therapeutic classes were considered, including antidepressants, antipsychotics, mood stabilizers, and anxiolytics. Searches were first conducted by psychotropic class and subsequently by individual drugs. The most frequently prescribed psychotropic drugs within each class, as well as drugs with reported anti-suicidal effects, were prioritized. Each drug class was combined with the selected pharmacogenes and suicide-related terms using Boolean operators. The overall search logic was defined using the following Boolean structure (“antidepressants” OR “antipsychotics” OR “mood stabilizers” OR “anxiolytics”) AND (“*ABCB1*” OR “*CYP2D6*” OR “*CYP2C19*” OR “*SCARA5*” OR “*GHSR*” OR “*FKBP5*” OR “*SAT1*” OR “*CRHR1*” OR “*NR3C1*”) AND (“suicide” OR “suicide ideation” OR “suicidal behavior” OR “suicide attempt”).

### Selection Scheme

Around 708 articles were identified. After removal of articles for which the full text could not be obtained, duplicate publications, publications with insufficient information, studies focusing on unrelated genes, and animal studies were excluded, leaving approximately 100 articles. In this phase, reference lists of selected articles were also manually reviewed to identify additional relevant studies. The remaining articles underwent title and abstract screening to verify the relevance of their content to our research, resulting in approximately 60 articles selected for full-text review.

During the eligibility phase, full-text articles were assessed using predefined inclusion and exclusion criteria. Studies were included if they were original articles published in English between 2000 and the present, were conducted in a human population, evaluated genetic variants in relation to suicidal behavior and/or response to psychotropic drugs. All types of studies were included, from case-control studies, cohort studies, meta-analyses, systematic reviews, replication studies, and case reports.

Studies were excluded if they didn’t report specific genetic data, didn’t mention suicidal outcomes, involved only animal models, or inconsistencies were found in the rs variants reported throughout the article, especially in the order or omission of the numbers. One *in vitro* study using B-lymphoblastoid cell lines was retained due to its relevance. Following full-text evaluation, 26 articles met all inclusion criteria and were included in the final narrative synthesis.

## Results 

### Genes Involved Pharmacokinetics

#### Genes Involved in Drug Absorption and Distribution

The *ABCB1* gene, named ATP-binding cassette subfamily B member 1, is located on chromosome 7 at region q21.12 and encodes for the permeability glycoprotein (P-glycoprotein or P-gp) [15]. It plays a role in the first stage of pharmacokinetics, as it is located on the apical surfaces of intestinal epithelial cells. Also has a participation in distribution, acting as a unidirectional transmembrane efflux pump powered by adenosine triphosphate, expressed in the blood-brain barrier, thereby limiting the entry of toxic substances into the central nervous system [16].

Regarding drug absorption, the *C3435T* rs1045642 polymorphism has been associated with reduced expression of P-glycoprotein in the small intestine. Since clozapine is not a substrate of the brain glycoprotein transporter, it is proposed that the intestinal transporter may play a role in clozapine absorption [17]. Consequently, individuals homozygous for the *c.3435CC* genotype rs1045642 polymorphism may require higher doses of clozapine [18].

In drug distribution, this gene has been associated with antipsychotics like aripiprazole, where people who are homozygous for the *T *allele of the single nucleotide polymorphisms (SNPs) *C1236T* rs1128503, *G2677T* rs2032582, and *C3435T* rs1045642 of the *ABCB1* gene tend to have lower drug concentrations [19].

As for antidepressants, a study in Japanese patients with MDD found that the haplotype combination *3435C–2677G–1236T* of the rs1128503-rs2032582-rs1045642 polymorphism of the *ABCB1* gene was associated with more severe depressive symptoms at six weeks of treatment with paroxetine [20]. Research also exists for antiepileptic drugs, which can be used as mood stabilizers. In Chinese children with epilepsy treated with valproic acid (VPA), those with the* TT* genotype rs1128503 may persist with seizures after treatment [21] (Table 1, Ref. [18, 19, 20, 21, 22, 23, 24, 25, 26, 27]).

**Table 1.** **Genes involved in pharmacokinetics**.

Drug involved	Variants rs	Effect	Reference
Genes involved in drug absorption and distribution			
*ABCB1*			
Clozapine	rs1045642	Higher dose required to reach target plasma concentrations.	Consoli *et al*., 2009 [18]
Aripiprazole	rs1128503	Lower concentration of the drug.	Toja-Camba *et al*., 2025 [19]
	rs2032582		
	rs1045642		
Paroxetine	rs1128503	Poor response due to more severe depressive symptoms at follow-up.	Kato *et al*., 2008 [20]
	rs2032582		
	rs1045642		
Valproic acid	rs1128503	*TT *genotype more likely to present persistent seizures after treatment.	Zhu *et al*., 2023 [21]
Genes involved in drug metabolism			
Cytochrome P450 family 2 subfamily D member 6 gene (*CYP2D6*)			
Clomipramine	rs1080985	Need for higher doses of the drug.	Antoniazzi *et al*., 2017 [22]
Paroxetine Vortioxetine	–	Ultra-rapid metabolism resulting in lower plasma concentrations of the drug.	Bousman *et al*., 2023 [23]
Duloxetine	rs3892097	Allele *A* presents a lower level of drug equilibrium concentration.	Zastrozhin *et al*., 2020 [24]
Valproic acid	rs3892097	2.5 times more likely to have treatment failure in patients with epilepsy.	Yazbeck *et al*., 2024 [26]
Cytochrome P450 family 2 subfamily C member 19 gene (*CYP2C19*)			
Citalopram Escitalopram	rs12248560	Rapid and ultra-rapid metabolism resulting in lower plasma concentrations of the drug.	Bousman *et al*., 2023 [23]
Diazepam	rs12248560	Reduced response in patients with alcohol withdrawal.	Skryabin *et al*., 2020 [25]
Genes involved in drug excretion			
*ABCB1*			
Dehydro-aripiprazole	rs1045642	Lower AUC and Cmax.	Saiz-Rodríguez *et al*., 2018 [27]
Risperidone	rs1045642	Lower AUC, Cmax and T½ and higher Cl/F.	Saiz-Rodríguez *et al*., 2018 [27]
9-OH-risperidone	rs1045642	Lower T½ levels.	Saiz-Rodríguez *et al*., 2018 [27]

This table contains a summary of the articles included in this review of the rs variants of the pharmacogenes that are related to pharmacokinetics, which involves from the administration of the drug and the achievement of drug concentrations throughout the body, and is divided in absorption, distribution, metabolism, and excretion. AUC, area under the curve; Cmax, maximum concentration; T½, half-life; Cl/F, higher apparent oral clearance.

This is relevant to suicidal behavior, as certain polymorphisms have been linked to a higher frequency of suicide attempts using violent methods among carriers of the haplotype *1236TT-2677TT-3435TT* of the rs1128503-rs2032582-rs1045642 polymorphism of the *ABCB1* gene [28], and to violent suicides among men carrying variants rs1128503, rs2032582 and rs1045642 of the *ABCB1* gene [29] (Table 2, Ref. [28, 29, 30, 31]).

**Table 2.** **Risk/Protective genes involved in pharmacokinetics and suicidal behavior**.

Gene	Suicide attempt	Completed suicide	Reference
*ABCB1*	Haplotype *TT* rs1128503-*TT* rs2032582-*TT* rs1045642 associated with higher frequency of using violent methods.	rs1128503, rs2032582, rs1045642 associated with higher frequency of violent suicides among men carrying at least one *T* allele.	Peñas-Lledó *et al*., 2015 [28] Boiso *et al*., 2013 [29]
*CYP2D6*	Increased risk of lifetime suicide attempt in patients with schizophrenia.	Higher number of UM among those who died by suicide.	Korchia *et al*., 2024 [31] Zackrisson *et al*., 2010 [30]
*CYP2C19*	Increased risk of lifetime suicide attempt in patients with schizophrenia and rs12248560 variant.	–	Korchia *et al*., 2024 [31]

This table contains a summary of variants and genotypes involved in pharmacokinetics and suicide behavior so the genetic associations can be visualized across the distinct phenotypes. UM, ultra-rapid metabolism.

#### Genes Involved in Drug Metabolism

The function of the cytochrome P450 enzyme system is to convert an ingested drug into a product that enters the bloodstream. The cytochrome P450 family 2 subfamily C member 19 gene (*CYP2C19*) is in the long arm of chromosome 10 at 10q23.33, which encodes the CP2CJ protein [32]; and the *CYP2D6* gene, involving subfamily D and member 6, is located on chromosome 22 at 22q13.2, which encodes the CP2D6 protein [33]. The increase activity of these enzymes, whether rapid (RM) or ultra-rapid metabolism (UM) can impact the metabolism of psychotropic drugs.

In the matter of antidepressants, the variant rs1080985 of *CYP2D6* gene has been studied in a case report involving clomipramine, showing high enzymatic activity [22]. Low or undetectable plasma concentrations of paroxetine and vortioxetine have been reported in *CYP2D6* UM, as well as significantly lower exposure to citalopram and escitalopram in *CYP2C19 *UM [23]. For duloxetine, men with MDD and mental/behavioral disorders due to alcohol use carrying the rs3892097 *A* allele of *CYP2D6* gene showed lower level of drug equilibrium concentration, which may affect efficacy [24].

With benzodiazepines, the rs12248560 polymorphism in genotypes *1/*17and *17/*17 of *CYP2C19* was associated with smaller score differences in the Clinical Institute Withdrawal Assessment scale before and after diazepam treatment in patients undergoing alcohol withdrawal [25]. In patients with epilepsy under treatment with valproic acid, the presence of rs3892097 of *CYP2D6* has linked to 2.5 more times of failure of the treatment [26] (Table 1). In relation to suicidal behavior, high *CYP2D6* activity has been linked to death by suicide in Swedish patients [30]. UM of *CYP2D6* and *CYP2C19* in French patients with schizophrenia have been associated with a higher lifetime risk of suicide attempts [31] (Table 2).

#### Genes Involved in Drug Excretion

The *ABCB1* gene plays a role in drug excretion and certain variants affect antipsychotic drugs; for example, individuals with the *T/T* genotype of the *C3435T* variant rs1045642 polymorphism exhibit lower values for area under the curve (AUC) and maximum concentration (Cmax) of dehydroaripiprazole. Also, this variant shows lower AUC, Cmax, and half-life (T½), and higher apparent oral clearance (Cl/F) of risperidone. The same genotype is also associated with a lower level of T½ for 9-OH-risperidone [27] (Table 1). In Table 3, are the variants and genotypes of the pharmacokinetic genes involved in altered response to psychotropic drugs so the genetic associations can be visualized across the different drug groups.

**Table 3.** **Variants and genotypes of the pharmacokinetic genes involved in altered response to treatment**.

Gene and variant	Antipsychotics	Antidepressants	Mood stabilizers or others
*ABCB1*	*TT* for aripiprazole and dehydro-aripiprazole.	Haplotype *C-G-T* for paroxetine.	–
rs1045642	*CC* for clozapine.		
	*TT* for risperidone and 9-OH-risperidone.		
*ABCB1*	*TT* for aripiprazole.		–
rs2032582			
*ABCB1*	*TT* for aripiprazole.		*TT* for valproic acid.
rs1128503			
*CYP2C19*	–	UM for citalopram and escitalopram.	UM for diazepam.
12248560			
*CYP2D6*	–	Increased metabolism for clomipramine.	
rs1080985			
*CYP2D6*	–	*A* allele for duloxetine.	Not specified for valproic acid.
rs3892097			

In the last column we added the “others” group to be able to add the finding of the benzodiazepine.

### Pharmacodynamics of Drugs

#### Scavenger Receptor Class A Member 5 Gene (*SCARA5*)

The *SCARA5* gene, or Scavenger Receptor Class A Member 5, is located on chromosome 8p21.1 [34]. It is a member of a membrane receptors family that can internalize a wide range of ligands and pathogens. Class A scavenger receptors are expressed in tissue macrophages, high endothelial venules, and certain dendritic cell subpopulations. Type 5 is specifically expressed in epithelial cells of the airways, the adrenal gland and the thymus, where it functions in bacterial binding and microbial defense [35].

The class A scavenger receptors (SR-As) are key pattern-recognition receptors expressed on activated microglia, particularly under pathological conditions associated with neuroinflammation. While these receptors are absent or minimally expressed in quiescent adult microglia, their upregulation has been observed in response to neuronal injury, infection, and inflammation [36]. By recognizing pathogen and damage associated molecular patterns, SR-As initiate and amplify inflammatory signaling cascades, promoting microglial activation and cytokine release [35]. Studies have reported that microglia participate in pathways altered upon suicidal behaviors: (a) as a result of susceptibility to stress, inflammation in the periphery may contribute to the failure of the blood-brain barrier and infiltration of inflammatory components could affect the regulation of microglial synaptic plasticity and (b) risk factors for suicidal behavior are associated with microglial priming [37]. The rs2685393 variant of *SCARA5* has been associated with suicide attempts in the Mexican population [11] (Table 4, Ref. [11, 38, 39, 40, 41, 42, 43]).

**Table 4.** **Risk/Protective genes involved in pharmacodynamics and suicidal behavior**.

Gene	Suicide attempt	Completed suicide	Probable decrease in the risk	Reference
*SCARA5*	Genotype *A1 C/A2 T* of rs2685393 variant associated with suicide attempt in the Mexican population.	–	–	González-Castro *et al*., 2019 [11]
*GHSR*	Genotype *A1 G/A2 T* of rs565105 variant associated with suicide attempt in the Mexican population.	–	–	González-Castro *et al*., 2019 [11]
*FKBP5*	Increased risk of suicide attempt in men with the *T* allele of the rs1360780 variant.	*TC* haplotype—rs1360780 and rs3800373—is related to completed suicide.	*CC* genotype of rs3800373 variant associated with positive association only in the female group.	Hernández-Díaz *et al*., 2021 [39] Fudalej *et al*., 2015 [38] Hernández-Díaz *et al*., 2021 [39]
*SAT1*	Association of the *C* allele of rs6526342 variant with suicide attempts in men.	–	Allele *A* of rs6526342 variant associated with increased expression of the gene.	Sokolowski *et al*., 2013 [40] Sequeira *et al*., 2006 [41]
*CRHR1*	Suicide attempts in men diagnosed with major depressive disorder associated with rs16940665 variant.	–	–	Pawlak *et al*., 2016 [42]
*NR3C1*	–	–	Probable decreased risk of attempt in patients with schizophrenia associated with rs6196 variant.	De Luca *et al*., 2010 [43]

This table contains a summary of variants and genotypes involved in pharmacodynamics and suicide behavior so the genetic associations can be visualized across the distinct phenotypes, being suicide attempt and completion and a phenotype of probable decrease in the risk of this behavior. Growth Hormone Secretagogue Receptor Gene (*GHSR*), FKBP Prolyl Isomerase 5 Gene (*FKBP5*), Spermidine/Spermine N1-Acetyltransferase 1 Gene (*SAT1*), Corticotropin Releasing Hormone Receptor 1 (*CRHR1*), Nuclear Receptor Subfamily 3 Group C Member 1 (*NR3C1*).

#### Growth Hormone Secretagogue Receptor Gene (*GHSR*)

The *GHSR* gene, or Growth Hormone Secretagogue Receptor, is located on the long arm of chromosome 3 in region 26.31 [44]. It is a G protein-coupled receptor, and its type 1a (GHSR-1a) has the ability to dimerize with other receptors such as dopamine D1 and D2, serotonin 2C (5-HT2C), cannabinoid receptor type 1, orexin, and melanocortin 3. GHSR-1a is expressed in the hypothalamus and in dopaminergic mesencephalic nuclei such as the ventral tegmental area and the compact part of the substantia nigra. In these limbic regions, it enhances dopaminergic neuron activation and increases dopamine release in the posterior striatum, which in turn heightens locomotion and reward-seeking behavior [45]. In the Mexican population, the rs565105 polymorphism of the *GHSR* gene has been linked to be associated with suicide attempts [11] (Table 4).

#### FKBP Prolyl Isomerase 5 Gene (*FKBP5*)

The* FKBP5* gene named by FKBP Prolyl Isomerase 5, is located on chromosome 6p21.31, which encodes for the intracellular FK506-binding protein that controls the sensitivity of the glucocorticoid receptor (GR) to cortisol [46]. Overexpression of the *FKBP5* gene affects the transcriptional activity of genes controlled by the steroid hormone signaling pathway in the HPA axis by decreasing the glucocorticoid receptor’s nuclear translocation and cortisol-binding affinity [47].

Drug response have been associated with SNPs in this gene, including a 2.11 fold increased risk of non-response to clozapine in people homozygous for the *T* allele of rs1360780 [48], and poor response to citalopram and escitalopram when the *A *allele of rs9380524 is present [49] (in Table 5, Ref. [48, 49, 50, 51, 52]). Regarding completed suicide, a Polish population study found an association between the *CC* and *CA* genotypes of the rs3800373 polymorphism in *FKBP5* and a relationship with the *TC* haplotype comprising the SNPs rs1360780 *T* and rs3800373 *C* [38]. As for suicide attempts, in a Mexican population specifically among men, the *T* allele of rs1360780 of *FKBP5* was correlated with increased risk. Additionally, the rs3800373 *CC *genotype was noted to have a protective effect in women [39] (Table 4).

**Table 5.** **Genes involved in pharmacodynamics**.

Drug involved	Variants rs	Effect	Reference
*FKBP5*			
Clozapine	rs1360780	2.11 fold increased risk of non-response.	Mitjans *et al*., 2015 [48]
Escitalopram	rs9380524	Poor response in patients with MDD.	Ellsworth *et al*., 2013 [49]
Citalopram			
*SAT1*			
Lithium	–	Increased *SAT1* mRNA levels were observed in patients with BD at risk of suicide. No effect was observed on completed suicide.	Squassina *et al*., 2013 [50]
*CRHR1*			
Fluoxetine	rs1876828	*GGT* carriers show poor response to fluoxetine in the high anxiety group.	Liu *et al*., 2007 [51]
	rs242939		
	rs242941		
*NR3C1*			
Escitalopram Nortriptyline	rs10052957	They were associated with response to antidepressants.	Uher *et al*., 2009 [52]
	rs10482633		
	rs852977		

This table contains a summary of the articles included in this review of the rs variants of the pharmacogenes that are related to pharmacodynamics, which involves the drug reaching its site of action and the onset, magnitude, and duration of the biological response. MDD, major depressive disorder; BD, bipolar disorder; mRNA, messenger RNA.

#### Spermidine/Spermine N1-Acetyltransferase 1 Gene (*SAT1*)

The *SAT1* gene, named spermidine/spermine N1-acetyltransferase 1, is located on the X chromosome at position Xp22.1. Its function is to regulate the polyamine system by adding acetyl groups to the aminopropyl ends of spermidine and spermine which favors its elimination and reduction of activity [53]. Polyamines influence in some neurotransmitters by regulating ionic flows binding to N-methyl-D-aspartate receptor where it prevents the release of magnesium from the channel, blocking AMPA receptors in the GABA system, and interacting with nicotinic receptors. Additionally, they play a role in the stress response, after a stressor a cascade of second messenger systems activates the polyamine stress response system (PSR) leading to rapid and transient increases in polyamine metabolism [54].

This gene has also been linked to lithium, has been observed a block in the brain’s PSR after lithium administration either before or after a stressful stimulus [55]. Furthermore, in an *in vitro* study on the effect of lithium treatment on *SAT1 *gene and protein expression in B lymphoblastoid cell lines from patients with BD, lithium significantly increased *SAT1 *messenger RNA levels in both low and high suicide risk BD groups but had no effect in those who had completed suicide [50] (in Table 5). In respect of suicide attempts, a study in Ukraine reported an association between the *C* allele of the rs6526342 polymorphism [40]. On the other hand, this pharmacogene has been reported as protective since the *A* allele of the SSAT342A variant rs6526342 polymorphism has been associated with greater expression of the *SAT1* gene in men of French-Canadian origin [41] (Table 4).

Box 1 **Polyamine stress response (PSR) system.**

The polyamine system is composed of putrescine, cadaverine, spermidine, and spermine performs various functions such as packaging nucleic acids, modulating membrane receptors and ion channels, and regulating gene expression and cell signaling. Three enzymes are required for the regulation of this pathway: ornithine decarboxylase, S-adenosylmethionine decarboxylase, and spermidine/spermine N1-acetyl transferase. The stressful stimulus that activates the PSR results in elevated levels of putrescine and agmatine. The magnitude of the response is related to the intensity of the stressor and correlates with the individual’s response pattern. This system can be manipulated pharmacologically, as in the case of lithium, which increases the expression of the *SAT1* gene and improves the response to stress. On the other hand, a polyamine depletion can produce altered emotional reactivity [56].

#### Corticotropin Releasing Hormone Receptor 1 (*CRHR1*) and Nuclear Receptor Subfamily 3 Group C Member 1 (*NR3C1*)

The HPA system is activated in response to a stressor, being the first step in the paraventricular nucleus of the hypothalamus where corticotropin-releasing hormone (CRH) is secreted, which binds to the corticotropin-releasing hormone receptor 1 (*CRHR1*), located on chromosome 17q21.31 [47] to the final step in the glucocorticoid receptors which are encoded by the *NR3C1* gene, named nuclear receptor subfamily 3 group C member 1, located on chromosome 5q31.3 [57].

There is also a relationship between the *CRHR1* gene and antidepressant response. Specifically for fluoxetine, in a Chinese population, carriers of the *T* allele of rs242941 showed a weaker response. Among patients with high-severity anxiety, those with the *GGT* haplotype rs1876828, rs242939, and rs242941, also had a deficient response to the drug [51] (Table 5). The rs16940665 variant in this gene has been associated with suicide attempts in men with MDD [42] (Table 4).

Lastly, the *NR3C1* gene appears to play a potentially protective role. The rs6196 variant has been associated with reduced risk of suicide attempts [43] (Table 4), and other variants rs10052957, rs10482633, and rs852977 have been associated with antidepressant response, particularly to escitalopram or nortriptyline [52] (Table 5). In Table 6, are the variants and genotypes of the pharmacodynamic genes involved in altered response to psychotropic drugs so the genetic associations can be visualized across the different drug groups.

**Table 6.** **Variants and genotypes of the pharmacodynamic genes involved in altered response to treatment**.

Gene and variant	Antipsychotics	Antidepressants	Mood stabilizers or others
*FKBP5*	*TT* for clozapine	–	–
rs1360780			
*FKBP5*	–	*A* allele for citalopram and escitalopram.	–
rs9380524			
*CRHR1*	–	Haplotype *GGT* for fluoxetine.	–
rs1876828			
rs242939			
rs242941			
*NR3C1*	–	Not specified for escitalopram and nortriptyline*.	–
rs10052957			
rs10482633			
rs852977			

*This is the only one related to an adequate response to antidepressants.

## Discussion

**Fig. 2.**
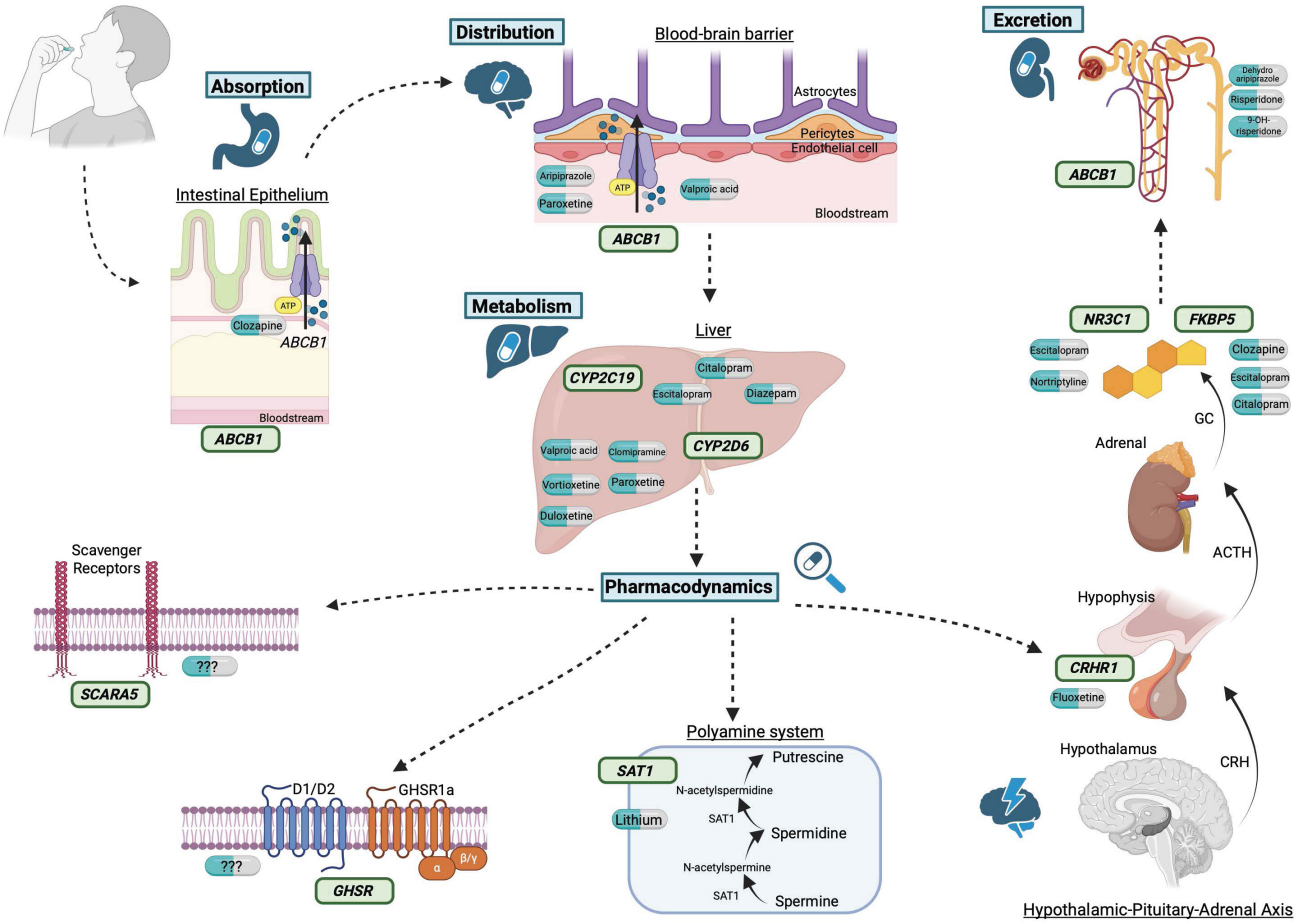
**Pharmacogenes involved in suicidal behavior in a pharmacokinetic and pharmacodynamic pathway**. When we take a medication, the first step in pharmacokinetics is absorption, where the *ABCB1* gene plays a role, specifically with the take of clozapine and the participation of intestinal transporter for adequate absorption. In distribution, the *ABCB1* gene again participates, but by presenting a variant, the distribution of selective serotonin reuptake inhibitors (SSRIs), atypical antipsychotics, and mood stabilizers would be altered. Upon entering the liver, variants in cytochromes can alter the metabolism of SSRIs and tricyclic antidepressants in the case of the two cytochromes, *CYP2D6* and *CYP2C19*; of benzodiazepines by *CYP2C19*; and of mood stabilizers, serotonin and norepinephrine reuptake inhibitors, and vortioxetine by *CYP2D6*. Turning to pharmacodynamics, *SAT1* gene is related to lithium, a mood stabilizer with anti-suicidal properties. Several genes are implicated in the hypothalamic-pituitary-adrenal axis, such as the *CRHR1* gene and its role in antidepressants, and the *FKBP5* gene and its involvement with the intake of clozapine and SSRIs. In the case of the *NR3C1* gene, variants in this gene are associated with response to escitalopram and nortriptyline. Regarding the *SCARA5* and *GHSR* genes, there is no information on alterations in the pharmacodynamics of psychotropic drugs. The last step in pharmacokinetics is excretion, where the *ABCB1* gene reappears and its role with atypical antipsychotics. Author: J.A.B.L. Created in BioRender. becerra, a. (2025) https://BioRender.com/l0r9n5n.

Since the publication of the first GWAS, research into genes involved in suicidal behavior has steadily increased, with a focus on physiological and biomarker perspectives. However, studying this subject is challenging due to the wide range of severity it encompasses. When investigating the pharmacogenes described above, we observe that following a pharmacokinetic and pharmacodynamic pathway results in several drugs being repeated, which can lead to a higher risk of non-response. For example, when taking clozapine, variants in the *ABCB1* and *FKBP5* genes must be considered; and antidepressants are of greater importance, as they are implicated in most of the pharmacogenes studied (Fig. 2).

One of the most studied pharmacogenes is the *ABCB1* gene and is involved in three steps of pharmacokinetics: absorption, distribution, and elimination; and associated with the response to drugs with anti-suicidal, antidepressant, antipsychotic, and mood-stabilizing properties.

The presence of the *T* allele is the most frequently observed in variants of *ABCB1* gene, in the case of aripiprazole it should be noted that the study was conducted with a monthly application of the drug using a long-acting injectable form, thus avoiding the participation of the transporter at the intestinal level and only evaluating at the P-gp of the blood-brain barrier, mentioning that the difference was not significant but a clear trend was observed and that one of the limitations was that they studied 72 patients [19]. On the other hand, when reporting variables that are related to elimination, significance is observed in a lower total exposure and a lower maximum peak concentration of dehydro-aripiprazole and a significant value towards a rapid elimination of 9-OH-risperidone but these findings are the result of the evaluation of the administration of a single dose and there is no long-term evaluation, although they emphasize that with this method confounding factors such as concomitant treatments are eliminated [27].

In the case of VPA, due to the frequency of the *TT* genotype it was suggested that it could be associated with a lower response, but it is important to mention that the SNPs was not associated with differences in drug concentration [21]. This is important in clinical practice since when starting it as a mood stabilizer valproic levels are requested for follow-up and if the patient presented this genotype we could be in a scenario of not obtaining a response, but a dose adjustment would not be required due to this SNPs and the best option would be a change in treatment. Regarding paroxetine, they report that the presence of the haplotype could be the cause of a poor response, and the favorable aspect of the study is that they only used this medication and the patients who were in another treatment but had no response were given 10 days for the total elimination of the previous drug. They highlight the lack of control of possible clinical confounding factors which in most cases are present in patients with psychiatric disorders since in the case of suicidal behavior there could be a mood or psychotic disorder [20].

In suicidal behavior, three variants have been identified which are repeated in both, attempted and completed suicide, which are 1236*C>T *rs1128503, 2677*G>T/A *rs2032582 and 3435*C>T *rs1045642 of the *ABCB1* gene, emphasizing that in both it was associated with violent methods. The difference is that in the attempt it was identified as a *TT-TT-TT *haplotype [28] and in suicide it is reported as at least one *T* allele in some of the variants [29]. In addition to the presence of the haplotype, they did an analysis only in women and obtained an odds ratio of 3.6 in using a violent method when committing an attempt, bringing it to the literature is important since we know that men use more violent and lethal methods unlike women. Studying populations that have committed suicide is more difficult due to limited data and samples, but in the study by Boiso Moreno *et al*. [29], they highlight as a strength that they studied almost 1000 postmortem cases although they did not have access to the medical records to control other variables.

In the case of cytochromes, the increased metabolism is the one that plays a role in both, response to drugs and suicidal behavior. In the *CYP2D6* analysis we found a case report detailing treatment failure at standard doses of clomipramine. After several attempts with other drugs, performing plasma levels of clomipramine and pharmacogenetic analysis using the Clinical pharmacogenetics implementation consortium (CPIC) guideline, it was increased to a maintenance dose higher than the one usually suggested, with which the patient obtained a response. The article only mentions that the pharmacogenetic result is heterozygous for the CYP2C19*17 promoter variant and *CYP2D6* rs1080985 but doesn’t perform analysis if the treatment failure is due to the combination of both cytochromes and doesn’t detail limitations in their report [22].

Other researchers report the lack of efficacy of duloxetine when analyzing the drug’s equilibrium concentration levels, which is relevant since this measure allows to evaluate the moment in which the speed of administration and elimination of the drug are in equilibrium; and for the rs3892097 variant it is significant, but the article also fails to present limitations in its research [24]. Again, we have evidence of VPA acting as an anticonvulsant, where the result of treatment failure linked to rs3892097 polymorphism is significant and this was adjusted to the maintenance dose of VPA. This is a strength since it contributes to the fact that the observed association between the genetic polymorphism and treatment failure is not influenced by differences in dosage. Although they mention that it is suggested to perform a population pharmacokinetic model to estimate VPA clearance for each individual since their population vary from 6 months to 40 years [26].

In cytochrome *CYP2C19*, the variant associated with increased metabolism is rs12248560, and in this pharmacogene we found the only evidence linked to benzodiazepines. Although its major limitation is the number of participants, it is a variant to be considered since the association was a lack of response to diazepam in patients with alcohol withdrawal [25], remembering that substance use is a risk factor for suicidal behavior. In the CPIC guidelines, we found that increased metabolism of both cytochromes affects the efficacy of antidepressants, but it must be considered that these are only recommendations for better clinical practice [23], and to date, no prospective studies have critically examined whether implementing such guidelines translates into reduced suicidal behavior.

The *CYP2C19* variant is the only one that is repeated between suicidal behavior and non-responsiveness to psychotropic drugs, since *CYP2D6* studies only report findings related to increased metabolism and do not specify a variant. In the study by Korchia *et al*. [31], they report an odds ratio of 4.096 for increased risk for lifetime suicide attempt in *CYP2D6* UM type and a risk of 2.680 for *CYP2C19* UM type, emphasizing that depressive symptoms and treatments did not influence this relationship. They also highlight that despite a large sample size, only 9% and 24.7% of the sample corresponded to UM metabolizers for *CYP2D6* and *CYP2C19*, respectively. In the research of completed suicide, it is emphasized that the research was carried out with different subtypes of individuals such as individuals who died of intoxication, of suicide, and of natural death, which contributes to a better analysis, but more information is needed about its limitations when carrying out the research. Furthermore, it is necessary to consider ethnic origin, since the frequency of *CYP2D6* duplications vary among populations [30].

Unfortunately, we did not find information on lack of response to psychotropic drugs related to the *SCARA5* and *GHSR* genes, and regarding the findings with suicide attempts in the Mexican population, the scavenger receptor gene is significantly represented and the *GHSR *gene is only statistically compared before applying the Bonferroni correction. Its main limitation is the participation of 37 cases and 155 controls, and they emphasize the need to consider the characteristics of suicide attempt phenotypes [11]. *SCARA5 *gene has more evidence of its linkage with suicidal behavior because of the different pathways in neuroinflammation and we believe that the lack of information on psychotropic drugs falls in its indirect role in neuroinflammatory pathways. Since decreased serotonin levels have been observed in individuals with depression because activated microglia increase the metabolism of tryptophan to quinolinic acid via the kynurenine pathway, and recent research has shown that this can also affect suicidal tendencies regardless of the affective diagnosis. Furthermore, this also affects the metabolism of kynurenic acid, which is a neuroprotective agent that inhibits glutamate, potentially leading to dysregulation and disruption of the blood-brain barrier. Additionally, quinolinic acid is a pro-oxidant and worsens the neurotoxic effects of corticosterone and pro-inflammatory cytokines [58]. Besides, it isn’t common for pharmacological studies to measure quinolinic acid or kynurenic acid so, future studies could take in account the kynurenine pathway to study the participation of *SCARA5* gene in psychotropic drug response.

Unlike the *GHSR *gene where the association isn’t clear and the fact that psychotropic drugs don’t directly target the ghrelin receptor of this gene could be the reason of lack of information. It has been proposed the participation of the *GHSR* gene in risk factors such as substance use and schizophrenia. First, it is suggested that *GHSR* antagonism reduces the reinforcing effects of substance use by reducing D1 receptor signaling produced by the D1R-GHSR1a dimer. Second, it has been shown that treatment with a *GHSR* inverse agonist improves intracellular 5-HT2C receptor signaling, which could suggest that it may increase the efficacy of 5-HT2C in the treatment of schizophrenia [59]. Future studies could combine *GHSR* modulators with psychotropic treatments to understand the changes induced by dimerization and the impact in treatment.

Regarding the pharmacogenes involved in stress pathways, we first analyzed the stress response linked to polyamines. Research has shown that lithium intake increases the expression of the *SAT1* gene and therefore adequate stress management. We still do not have information of a polymorphism on this pharmacogene that has been related to the lack of response to lithium, but we know that the expression of this gene is not modified in patients who commit suicide [50]. In the findings of suicidal behavior, the rs6526342 variant is associated with suicide attempt when the *C *allele is present [40]. It has also been reported a probable decrease in risk due to an increase in *SAT1 *gene expression when the *A* allele is present, these authors obtained brain samples from people who had committed suicide and emphasize the consistency of the significance of their results in the brain regions they studied and validated them using alternative (RT-PCR) and complementary methods (immunohistochemistry and Western blot) [41]. Therefore, we emphasize that both, the variables of each gene and the allele or genotype, that are present in the patients are of interest to identify a risk of suicidal behavior because decrease in *SAT1* expression could facilitate suicidal behavior and on the other hand, its increase may be linked to a protective role.

Secondly, in the HPA axis, the variants rs1876828, rs242939 and rs242941of the *CRHR1* gene are involved in the response to fluoxetine in a short-term treatment of 6 weeks. We highlight that this study considered the variable of anxiety, since in the literature the gene has been implicated in depression and anxiety, so they divided their sample into low and high anxiety groups. In the end, they conclude that the phenotype of the response to antidepressant treatment is heterogeneous because variations in the gene may be associated with these two disorders. They also mention that it would be necessary to measure plasma levels of fluoxetine for future research [51]. The variables analyzed above differ from the one found in the association with suicide attempt that is rs16940665, this finding remained significant after multiple testing correction and these authors emphasized that they didn’t consider psychological, social and clinical factors, the time of untreated disease or lack of compliance, which they suggest should be considered for future analyses [42].

When talking about glucocorticoids we have the participation of two genes, *FKBP5* and *NR3C1*. From the *FKBP5* gene, the most relevant finding is that the rs1360780 variant was found to be associated in three scenarios: (1) in lack of response to clozapine, a drug with anti-suicide properties, but the finding remained no longer significant after permutation analysis and the authors point out that the response assessment was performed retrospectively from medical notes, which could lead to inaccuracies [48]; (2) in suicide attempt, it has been associated in a statistically significant manner and which remained among men using three genetic models (*p* < 0.006) [39] and (3) in completed suicide with the presence of an haplotype with an odds ratio of 1.34 [38].

In contrast to cytochrome *CYP2C19*, the rs1360780 *T* and rs3800373 *C* haplotypes of the *FKBP5* gene were related to non-impulsive suicides because the individuals who committed the suicide were not under the influence of alcohol [38]. Furthermore, in this pharmacogene, we found the second variant associated with a probable decreased risk of suicidal behavior, which has been mentioned previously and it’s rs3800373 [39]. It should be noted that the finding was in a Mexican population and that the haplotype of risk was in a Polish population, so if it is replicated in Mexican population, this could serve as protective biomarker. Regarding the *NR3C1* gene, we found a third variant rs6196 that could offer protection in the population with schizophrenia. In this article, only 11 subjects presented this genotype with a *p* = 0.0363 and due to the small sample size of 81 patients who attempted suicide, the authors mention that the findings should be considered preliminary [43].

Box 2 **Dysregulation of the HPA axis and the *FKBP5* gene.**

In normal functioning, the hypothalamic-pituitary-adrenal system is activated when it detects a stressor, the paraventricular nucleus secretes CRH in the hypothalamus to bind to its receptor CRFR1, which is coupled to G proteins, in the anterior pituitary gland. This induces the release of adrenocorticotropic hormone (ACTH) in the pituitary gland and activation of the adrenal cortex to synthesize GR and mineralocorticoids. There are several mediators of the stress response, the corticotropin-releasing hormone-binding protein (CRHBP) that modulates the activation of CRH receptors in the brain and periphery and inactivates the circulation of CRH in the plasma, the activation of GR triggers a negative feedback loop that attenuates the axis and the FK506 binding protein that regulates the sensitivity of the GR receptor [60].

It is proposed that risk variants are associated with increased *FKBP5* expression, reduced GR sensitivity, and impaired negative feedback of the HPA axis [38]. Specifically, the rs1360780 *T* allele apparently causes a difference in DNA conformation, interacting with the TATA box binding protein, which causes direct contact with the transcription start site and RNA polymerase II, resulting in transcriptional activation of *FKBP5* [39]. Therefore, we hypothesize that the alteration in the HPA axis combined with the lack of response to psychotropic drugs would increase the risk of presenting suicidal behaviors.

Regarding the variants that affect the axis, we focused on the anterior pituitary gland with the *CRHR1* gene and on the adrenal glands with the *NR3C1* gene. Post-mortem studies of suicide completers have visualized alterations in the axis by observing increased CRH activity in the paraventricular nucleus, fewer CRH binding sites in the frontal cortex, and decreased glucocorticoid receptor expression in the hippocampus [56]. Therefore, we hypothesize that if there are genetic alterations in the CRH receptor gene, it will trigger alterations in the axis and risk of suicidal behavior.

The *NR3C1* gene has been associated with the presence of childhood adversity, which is a risk factor for suicidal behavior. This gene was found downregulated in the hippocampus of individuals exposed to childhood adversity, which leads to poor regulation of the axis [56]. Therefore, finding variants that may have a protective effect in addition to finding variants that respond to drugs may be another side of the coin for this gene.

Talking about suicidal ideation, wasn’t until two years ago that the first GWAS focusing solely on suicidal ideation was conducted and in our search for literature we realized that in some studies, they used the ideation as an exclusion criterion, while others address suicidal behavior as a whole without distinguishing between phenotypes. Unfortunately, none of the pharmacogenes we studied were related to suicidal ideation, so further study is needed in this field.

With this narrative review, it is clear that various gene variants lead to reduced drug concentrations, lower exposure, shorter duration, and poor or no response, ultimately meaning that patients may not benefit from treatment. This can lead to persistent or worsening symptoms that may culminate in suicidal behavior. We also want to highlight that different variants of the studied pharmacogenes show a repetition across the phenotypes of suicidal behavior and even are presented in the altered response to psychotropic drugs.

For example, the variants rs1128503, rs2032582, and rs1045642 of the *ABCB1* gene are associated with suicide attempts, completed suicides, and altered responses to aripiprazole and paroxetine. In the case of the *FKBP5* gene, the rs1360780 variant is associated with suicide attempts, completed suicides, and altered responses to clozapine. Additionally, there are variants in the *FKBP5* and *SAT1* genes that are associated with suicidal behavior, and the same variant has been reported with a probable decrease in risk, being rs3800373 for the *FKBP5* gene and rs6526342 for the *SAT1* gene.

In addition to the above, we emphasize the precedent that the most recent GWAS on suicidal ideation replicated findings from the ISGC’s suicide attempt data, identifying *EXD3* and *ESR1* genes. Therefore, they suggested a genetic overlap between suicide phenotypes and provided a likely explanation for the loci that weren’t associated with suicide attempt in the ISGC, being that they might be specific to suicidal ideation rather than shared with suicide attempt [12].

Therefore, with this information and the findings of this review, we could hypothesize that there is a possibility of finding shared genetic mechanisms among suicide phenotypes. The information in this review is a preliminary, since, although there are repeated variants, we must remember that these are different studies with heterogeneous methodologies, populations, and diagnostic criteria. We propose that future research replicate the variants that may show a genetic association in the same population, evaluating the three suicide phenotypes to determine whether the apparent repetition of variants reflects a real genetic overlap or whether each phenotype presents a specific genetic profile.

Furthermore, the finding of repeated variants between suicidal behavior and altered response to psychotropic drugs is relevant information since these pharmacogenes participate in different functional pathways such as neurotransmission, HPA regulation and neuroinflammation and simultaneously can influence the efficacy of psychotropic treatment. So, we hypothesize, this combination could lead to a higher risk of suicidal behavior.

The information provided can give us an overview and encourage the study of suicidal behavior from a pharmacogenomic perspective to advance precision medicine. Pharmacogenomic tests currently available include genes for cytochromes, catecholamine metabolism, drug transport proteins, cytoskeleton, drug biotransformation, Human Leukocyte Antigen (HLA), and receptors for dopamine, glutamate, and serotonin. Also, a 2024 review stressed the need to standardize pharmacogenomic test panels due to their heterogeneity, as the benefits of PGx-guided antidepressant prescriptions may vary depending on the panel used [61].

So, the creation of new pharmacogenomic test panels or introducing other pharmacogenes or variants to the existing ones could be incorporated into the evaluation of patients with suicidal behavior to improve phenotype-based prescription. In particular, pharmacogenomic tests could be useful in those with prior lack of response, a history of adverse reactions, complex polypharmacy, patients with a structured suicide plan or those who are readmitted to medical care due to suicide. In these cases, testing could help inform treatment decisions by identifying early pharmacokinetic and pharmacodynamic alterations of medications that could be part of the therapeutic plan, thus reducing trial-and-error prescribing and potentially decreasing the risk of suicide by optimizing the response to medication.

In this context, with the concept of a potential therapeutic window used on the title, we refer to a clinically period in which pharmacogenomic information may be used to optimize psychotropic treatment decisions in patients with suicidal behavior, prior to treatment failure, adverse drug reactions, or further escalation of suicide risk. Further research is needed on the use of pharmacogenomic tests, as the FDA states that only a few genetic variants have sufficient scientific evidence and that the clinical impact hasn’t been evaluated. The associations they list include cytochromes, *HLA*, and other enzymes involved in metabolism [62].

Currently, as research continues to progress, a phased pharmacogenomic evaluation could be conducted. This could begin with pharmacogenes of greater clinical relevance already included in panels, such as cytochromes and the *ABCB1* gene. Based on these results, treatment could be initiated or dosages of prescribed medications adjusted. Simultaneously, exploratory pharmacogenomic testing could be performed, addressing the pharmacogenes mentioned in this review that are still under investigation but whose repeated association with suicidal behavior and treatment response suggests potential value in generating individualized hypotheses about treatment resistance, adverse outcomes, or an increased risk of suicidal behavior.

In another scenario, integrating pharmacogenomics into clinical practice could be observed in patients who come to the clinic with suicidal ideation, in whom, upon identifying that they present a rapid or ultra-rapid metabolizing phenotype, the choice of drug is reconsidered and perhaps not starting with a first-line antidepressant such as an SSRIs and switching to other groups. Ultimately, the goal is to provide safe and effective prescribing that offers patients timely symptom relief without requiring multiple treatment changes or prolonged waiting periods.

One recurring limitation across studies was the small sample size, which is understandable because stigma can play a role in identifying the true cause of death or that patients talk about their suicidal behaviors to healthcare professionals. Moreover, it is important to highlight the huge number of variables to consider for inclusion and exclusion in a study of suicidal behavior. In our opinion, future research might consider psychiatric comorbidities to analyze the association between the variants and suicidal behavior alone, and in conjunction with psychiatric diagnosis, in the matter of pharmacological treatment, consider initiation and follow-up including adherence and discontinuation so that the association with suicide isn’t biased because the patient didn’t take the prescribed treatment properly; and information of the suicidal behavior such as characteristics of previous attempts such as age at the first attempt and lethality, criteria that were used for the forensic autopsy and, if possible, characteristics of suicidal ideation, as well as completing the medical information of individuals who died by suicide, since suicide is a whole spectrum and its multiple phenotypes could be associated or generate bias.

One limitation of this review is population variability across studies, including research from Asia in Japan and China, Russia, Europe in Italy, France, Ukraine, Spain, Sweden, and Poland; and the Americas with Mexico, Canada, and U.S. The above is important because the frequency of alleles, haplotypes, and genotypes varies among populations, so risk variants found in one specific group can’t be generalized to another, and replication studies are necessary. For example, in the case of the C3435T polymorphism, the African population generally presents a CC genotype, unlike Caucasians and Asians, who are more frequently heterozygous [63].

Most research focuses on European populations, highlighting the need to include more Latin American cohorts for clinically applicable conclusions that can address the growing mental health crisis [64]. Another limitation is the diversity of psychiatric disorders analyzed, such as affective, psychotic, and anxiety disorders. One study even used healthy volunteers, reducing generalizability. Taken together, the observed variability across populations, psychiatric diagnoses, pharmacological exposures, and suicidal phenotypes reflects substantial clinical, genetic, and methodological heterogeneity. Given the narrative design of this review and the lack of sufficiently homogeneous data to allow quantitative pooling, formal statistical heterogeneity analysis weren’t performed. Instead, findings were interpreted through a qualitative, contextual synthesis aimed at identifying consistent patterns and generating hypotheses. Finally, this narrative review has a limitation related to its qualitative nature. Although an adapted PRISMA flow diagram was included in the methodology section, the literature selection process still relied largely on the authors interpretation and did not follow a fully systematic protocol for study inclusion or bias assessment.

## Conclusions

The reviewed literature highlights that research already exists on pharmacogenes related to suicidal behavior. However, more investigation is necessary, particularly to replicate findings in the Mexican population, so that these pharmacogenes might be considered for inclusion in existing pharmacogenomic testing panels. Moreover, certain variants are repeated across different suicidal behavior phenotypes and in altered response to different psychotropic drugs. These could provide a promising direction for further investigation in order to analyze if there is a genetic overlap or each phenotype presents a specific genetic profile. Despite the limitations mentioned, the information gathered provides a possible overview of the involvement of pharmacogenes in the functional pathways of suicidal behavior and thus the possibility of enriching future research. Finally, new publications continue to show improvements in patients who undergo PGx testing, hence, it is essential to pursue this area of research to address behaviors that pose a risk to life.

## Availability of Data and Materials

Not applicable.
